# 118 Burn Injury Vandalizes Cancer Survival with Increased Risk of Complications

**DOI:** 10.1093/jbcr/irac012.120

**Published:** 2022-03-23

**Authors:** Phillip H Keys, Sunny Gotewal, Kendall Wermine, Tsola A Efejuku, Shivan N Chokshi, Lyndon G Huang, Kassandra K Corona, Elvia L Villarreal, Jasmine M Chaij, Giovanna De La Tejera, Shelby P Bagby, Maria Haseem, Alejandro A Joglar, George Golovko, Steven E Wolf, Amina El Ayadi, Juquan Song

**Affiliations:** University of Texas Medical Branch, Galveston, Texas; University of Texas Medical Branch, Galveston, Texas; University of Texas Medical Branch, Galveston, Texas; University of Texas Medical Branch, Galveston, Texas; University of Texas Medical Branch at Galveston, Galveston, Texas; University of Texas Medical Branch, Galveston, Texas; University of Texas Medical Branch, Galveston, Texas; University of Texas Medical Branch, Galveston, Texas; University of Texas Medical Branch, Galveston, Texas; University of Texas Medical Branch, Galveston, Texas; University of Texas Medical Branch, Galveston, Texas; University of Texas Medical Branch, Galveston, Texas; University of Texas Medical Branch, Galveston, Texas; University of Texas Medical Branch at Galveston, Galveston, Texas; University of Texas Medical Branch at Galveston, Galveston, Texas; University of Texas Medical Branch at Galveston, Galveston, Texas; University of Texas Medical Branch, Galveston, Texas

## Abstract

**Introduction:**

Burn injuries place patients in a compromised state, especially those with pre-existing comorbidities. The presence of cancer complicates care and worsens outcomes for patients suffering from illnesses unrelated to burns, such as sepsis. Therefore, we posit the incidence of burn injury on patients with preexisting cancer diagnoses results in an increased risk of complications.

**Methods:**

Burned patients were identified using the TriNetX database, a global federated health research network. Fifty-one thousand patients with a diagnosis of cancer prior to experiencing a burn injury were identified. Control groups included 1) patients who had a previous cancer diagnosis and no incidence of burn, and 2) patients who experienced a burn with no history of cancer. Outcomes analyzed included sepsis, nutritional deficiency, eating disorder, immunodeficiency, and depression within 5 years. Cancer diagnoses were categorized into 5 of the 13 most common cancer reported in the US. Data was analyzed using a chi-square analysis with p< 0.05 considered significant, and presented odds ratio are with 95% confidence intervals.

**Results:**

The majority of cancer survivors with burns were White (70%) and female (62%). Compared to cancer patients without burn injury, patients experiencing a burn after a diagnosis of cancer were more likely to develop sepsis (1.718, 1.612-1.83), nutritional deficiency (1.963, 1.593-2.418), immunodeficiency (1.265, 1.098-1.459), eating disorders (2.569, 2.077-3.177), and depression (1.538,1.468-1.611). When compared to burn patients with no history of cancer, burned patients with cancer diagnosis had increased odds of developing sepsis (3.806, 3.502-4.137), nutritional deficiency (3.529, 2.725-4.571), immunodeficiency (6.657, 5.126,8.645), eating disorder (2.184, 1.787-2.67), and depression (2.147, 2.041-2.259). Further, burned patients with a history of lung cancer experienced a uniquely high risk of sepsis. Additionally, burn patients with histories of either lung or breast cancers were also at increased risk ratios of experiencing depression (p< 0.05).

**Conclusions:**

Burned patients with a history of a cancer demonstrated considerable increases in complications when compared to those with only a burn injury. Categorization of the broad “neoplasm” label uncovers patterns or trends for specific cancer types to inform the current healthcare system more accurately.

